# Multimodal neuroimaging discrimination of Alzheimer’s disease, mild cognitive impairment, and late-life depression using electroencephalography and functional near-infrared spectroscopy: integrating electrophysiological and hemodynamic biomarkers

**DOI:** 10.3389/fnagi.2026.1713472

**Published:** 2026-02-06

**Authors:** Xi Mei, Ming Liang, Nairong Ruan, Zheng Zhao, Ting Xu, Chengying Zheng

**Affiliations:** 1 Department of Psychiatry, Affiliated Kangning Hospital of Ningbo University, Ningbo, Zhejiang, China; 2 Department of Psychiatry, The Third People’s Hospital of Xiangshan County, Ningbo, Zhejiang, China; 3 Department of Psychiatry, Ningbo Psychiatric Hospital, Ningbo, Zhejiang, China

**Keywords:** Alzheimer’s disease, EEG, fNIRS, late-life depression, mild cognitive impairment

## Abstract

**Objective:**

To identify electrophysiological and hemodynamic characteristics of the cerebral cortex during the resting-state that could help differentiate Alzheimer’s disease (AD), mild cognitive impairment (MCI), and late-life depression (LLD) and integrate these characteristics into a diagnostic model.

**Methods:**

We recorded oxygenated hemoglobin concentration (HbO) signals detected by functional near-infrared spectroscopy (fNIRS) from the prefrontal cortex, partial parietal cortex, and temporal lobe cortex, as well as electrophysiological signals detected by electroencephalography (EEG). The recording time was 30 min. Then, we used machine learning modeling with the support vector machine (SVM) algorithm to evaluate the diagnostic performances of EEG-based, fNIRS-based, and EEG plus fNIRS-based models for distinguishing AD, MCI, and LLD.

**Results:**

We investigated the differential neural signatures of patients with AD (*n* = 61), MCI (*n* = 28), and LLD (*n* = 27) using an EEG power spectral analysis across six frequency bands: delta (1–4 Hz), theta (4–8 Hz), alpha (8–13 Hz), beta (13–30 Hz), low gamma (30–45 Hz), and high gamma (55–80 Hz). Two key frequency bands significantly differed among the groups. The alpha band power was significantly higher in the LLD group than in the AD and MCI groups (*p* < 0.05). The high gamma power was also significantly higher in the LLD group than in the MCI group (*p* < 0.05). Regarding fNIRS, HbO power significantly differed in 11 channels (channels 19, 22, 23, 24, 26, 27, 28, 32, 41, 42, and 43); the values were significantly lower in the AD group than in the MCI group and significantly higher in the MCI group than in the LLD group. The accuracies of the EEG, fNIRS, and combined SVM models were 0.5246, 0.5246, and 0.6066, respectively.

**Conclusion:**

These findings highlight distinct EEG spectral patterns in patients with LLD compared to those with AD or MCI, particularly in alpha and high-gamma oscillations. These differences could be potential biomarkers for differentiating these conditions. Combining EEG and fNIRS analyses may further elucidate the neurophysiological mechanisms underlying these disorders.

## 1 Introduction

Cognitive decline is a common clinical manifestation of age-related neurodegenerative or neuropsychiatric disorders, including Alzheimer’s disease (AD) and late-life depression (LLD) (Gupta et al., 2023). AD, a neurodegenerative disorder, and mild cognitive impairment (MCI), a neurocognitive disorder, are often accompanied by neuropsychiatric symptoms. However, their primary pathophysiology is distinct from primary psychiatric illnesses. Most patients with LLD have cognitive impairment, and at least one-third of them meet the diagnostic criteria for MCI, a prodrome of AD and other neurodegenerative diseases in older adults (Marawi et al., 2023). However, the mechanisms linking LLD, MCI, and AD with the underlying brain alterations that cause impaired cognition in age-related neurodegenerative diseases remain poorly understood.

Cognitive deficits associated with LLD can involve any cognitive domain. Previous studies have reported that the core cognitive deficits in patients with LLD include poor executive function and reduced information processing speed (Ainsworth et al., 2024; Dybedal et al., 2013). Other cognitive domains may also be affected by these two core cognitive deficits (Faoro and Hamdan, 2021; Sheline et al., 2006; Wen et al., 2022). Moreover, LLD is associated with an increased risk of dementia in patients with AD (Zhang et al., 2025). Compared to early-onset depression, LLD has been associated with more severe cognitive impairment, white matter hyperintensities, and other cerebrovascular abnormalities common in AD (Garnier-Crussard et al., 2023; Sachs-Ericsson et al., 2013; Shirzadi et al., 2023). Some studies have suggested that LLD is an early neuropsychiatric manifestation of AD.

However, the neurobiological mechanisms underlying LLD, MCI, and AD differ. The pathology of AD includes amyloid-beta accumulation in brain regions associated with impaired episodic memory (Hampel et al., 2021). The amyloid hypothesis of LLD suggests that patients with a lifetime history of depression have significant amyloid accumulation in brain regions related to mood regulation, which leads to cell inflammation, vascular damage, cerebral blood flow reduction, and disrupted functional connectivity that impairs the brain regions and networks implicated in mood or depression (Alexopoulos, 2019; Mahgoub and Alexopoulos, 2016). Previous clinical trials and reviews have comprehensively discussed the mechanisms linking LLD, MCI, and AD (Panza et al., 2023; Rajji et al., 2025; Taylor et al., 2022). That while theoretical links are discussed in the literature, there is a gap in direct, multimodal neurophysiological biomarkers for practical differentiation. That our study aims to fill this gap by identifying and integrating such biomarkers, thus providing an empirical bridge between known mechanistic theories and clinical application.

Several neuroimaging studies have identified structural alterations in the brain associated with LLD or MCI that contribute to the risk of AD (Anderson et al., 2024; Lebedeva et al., 2017; Rashidi-Ranjbar et al., 2020a; Rashidi-Ranjbar et al., 2020b). In addition to magnetic resonance imaging, brain activity can be detected using functional near-infrared spectroscopy (fNIRS), a noninvasive neuroimaging technique for investigating neurodegenerative or neuropsychiatric disorders (Albrecht et al., 2025; Erdoğan and Yükselen, 2022). It uses near-infrared light to detect temporal changes in cerebral cortical oxygenated hemoglobin (HbO) and deoxygenated hemoglobin (HbR) concentrations (Jöbsis, 1977). fNIRS is a safe and cost-effective technique with a robust anti-interference ability; thus, it is widely used to measure cerebral hemodynamics in patients with neuropsychiatric diseases during multiple cognitive tasks (Akın, 2021; Duan et al., 2025). fNIRS measures the concentrations of HbO and HbR to estimate the underlying cognitive processes of probe-covered cerebral cortices during different cognitive tasks and resting states (Mei et al., 2024; Yang et al., 2025; Yeung and Chan, 2020).

The accurate differential diagnosis of AD, MCI, and LLD remains a significant clinical challenge, primarily due to the overlap in their cognitive and neuropsychiatric presentations. A critical knowledge gap exists in the lack of robust, objective neurophysiological biomarkers derived from accessible technologies like EEG and fNIRS for this specific diagnostic triage. Therefore, the primary goal of this study was to identify distinct resting-state spectral EEG and fNIRS-based hemodynamic characteristics across these patient groups. By integrating these multimodal features within a support vector machine (SVM) framework, we aimed to develop a novel data-driven model to aid in distinguishing AD, MCI, and LLD, thereby addressing this unmet clinical need.

Neuroimaging provides a critical window into the neural correlates of neurological and psychiatric disorders (Zhao et al., 2025). However, single-modality approaches offer a limited perspective: EEG excels at capturing neural activity with millisecond temporal resolution but suffers from poor spatial resolution and limited depth penetration, making it difficult to localize signals to specific brain regions (Goerttler et al., 2024). fNIRS measures hemodynamic changes (like fMRI) with good spatial resolution on the cortical surface and is robust to motion artifacts, but its temporal resolution is limited by the slow hemodynamic response (seconds) (Sainbhi et al., 2023). Combining EEG and fNIRS is particularly powerful because it allows for the simultaneous mapping of electrical and hemodynamic brain activities. This synergy helps to paint a more complete picture of brain dysfunction. For instance, a disorder might be characterized by both: Aberrant neural timing or synchronization (detectable by EEG oscillations or event-related potentials); Dysregulation of metabolic demand in specific cortical areas (detectable by fNIRS via HbO/HbR concentration changes).

This study combined fNIRS with electroencephalography (EEG) to analyze cerebral cortex activation and functional connectivity during the resting state in older adults with different types of neuropsychiatric diseases. We also aimed to identify potential biomarkers of neuropsychiatric diseases and examine the performances of different diagnostic models based on the fNIRS and EEG biomarkers.

## 2 Materials and methods

### 2.1 Participants

**FIGURE 1:**
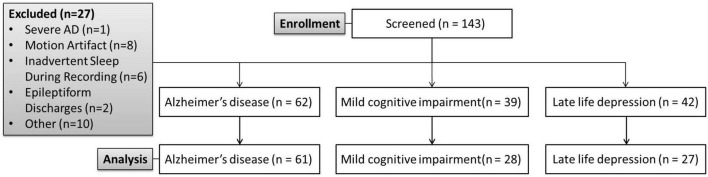
CONSORT diagram of the primary phases of the study.

We recruited patients with AD, MCI, and LLD from the Geriatric Psychiatric Center of the Affiliated Kangning Hospital of Ningbo University between February 2024 and March 2025 (Figure 1).

#### 2.1.1 Inclusion/exclusion criteria

All enrolled participants were diagnosed according to the fifth edition of the Diagnostic and Statistical Manual of Mental Disorders (DSM-5). The diagnostic process involved independent clinical assessments by two research psychiatrists, and a consensus diagnosis was reached for each participant. To be included in the study, participants were required to meet the following criteria: (1) a confirmed diagnosis of Alzheimer’s Disease (AD), Mild Cognitive Impairment (MCI), or Late-Life Depression (LLD); (2) a documented disease course exceeding 6 months; and (3) provision of written informed consent.

Participants were excluded from the final analysis based on predefined criteria aimed at ensuring data quality and homogeneity of the study sample. A total of 27 participants were excluded for the following reasons: severe AD (*n* = 1), excessive motion artifact during signal acquisition that compromised data quality (*n* = 8), inadvertent sleep during the neurophysiological recording session (*n* = 6), presence of epileptiform discharges in the recorded data (*n* = 2), and other reasons not directly related to the primary study protocol (e.g., technical failure, protocol deviation; *n* = 10). The final analyzed cohort thus comprised participants who met all inclusion criteria and none of the exclusion criteria.

### 2.2 Neuropsychiatric evaluations

Cognitive impairment was assessed using the Mini-Mental State Examination (MMSE), a 30-point screening tool for cognitive impairment. Scores < 11 often indicate severe dementia. The Hamilton depression scale (HAMD) was used to evaluate depression, with scores > 7 indicating depression. All participants underwent the MMSE and the HAMD assessment on the same day as the EEG and fNIRS recording session, immediately prior to the neurophysiological setup. This protocol ensured that the clinical scores were contemporaneous with the acquired brain signals, providing a direct correlation between the neuropsychological state and neurophysiological measures.

### 2.3 EEG examination and analyses

The participants were seated in a comfortable chair. The EEG recording was performed using a 128-channel EEG (EGI System 300; Electrical Geodesic Inc., Eugene, OR, United States) configured in the standard 10–20 montage with reference to the linked mastoids. The sampling rate was 500 Hz using an amplifier with low and high cut-off frequencies of 0.1 and 100 Hz, respectively.

EEG recordings were compiled during the resting state using a structured testing and acquisition software platform (5 min with the eyes open, and 5 min with the eyes closed). During the eyes-open task, participants were instructed to stare directly at a black fixation cross located at the center of a gray background. During the eyes-closed task, the participants were instructed to close their eyes while maintaining wakefulness. NetStation software (Electrical Geodesic Inc.) was used for the recording. The impedance for all electrodes was kept below 50 kΩ. Offline data analysis was performed using the open-source EEGLAB toolbox (Swartz Center for Computational Neuroscience, La Jolla, CA, United States).

#### 2.3.1 EEG power analysis

Resting-state eyes-closed and eyes-open EEG samples were analyzed using a fast Fourier transform approach. The power spectral density was calculated for six frequency bands: delta (1–4 Hz), theta (4–8 Hz), alpha (8–13 Hz), beta (13–30 Hz), low-gamma (30–45 Hz), and high-gamma (55–80 Hz). PSD (power spectral density) was calculated by the Welch’s averaged periodogram method. The power spectrum for each epoch was computed via Fast Fourier Transform (FFT), resulting in a frequency resolution of 0.5 Hz. PSD was integrated within six clinically relevant frequency bands to extract band-specific power.

#### 2.3.2 EEG weighted-phase lag index analysis

The EEG signals were transformed into the frequency domain, and then the phase differences between different brain regions at each frequency were calculated. Next, we computed the wPLI based on the statistical distribution of the phase differences. wPLI values range from 0 to 1, where 0 and 1 indicate no phase coupling and complete phase coupling, respectively (Vinck et al., 2011).

### 2.4 fNIRS measurement and processing

**FIGURE 2:**
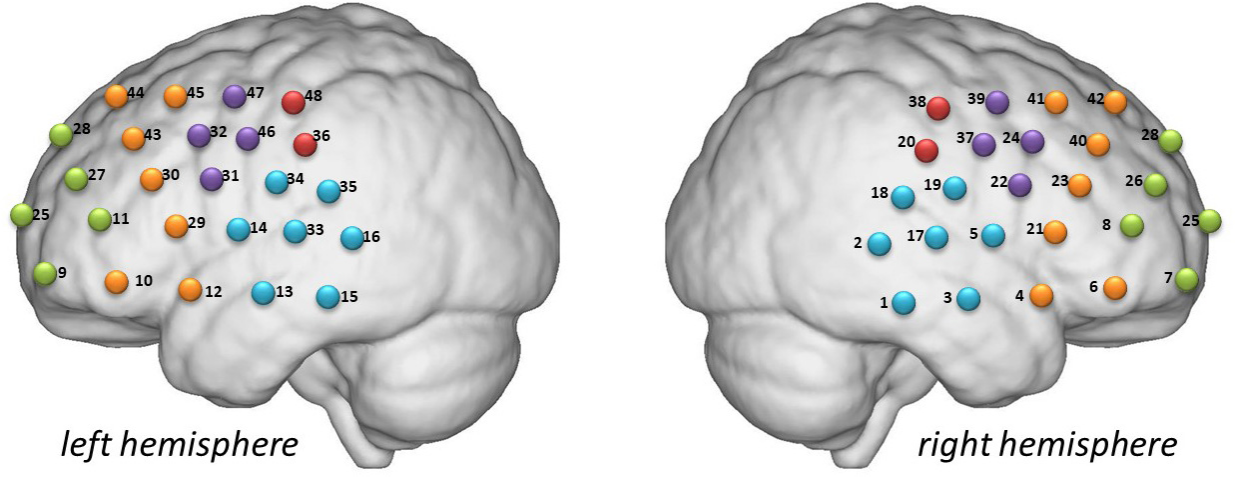
The channel locations of fNIRS measurement. The frontopolar area contained channel 7, 8, 9, 11, 25, 26, 27, and 28; the right pars triangularis Broca’s area contained channel 22, 24, 37, and 39; the right pre-motor and supplementary motor cortex contained channel 20 and 38; the right temporal cortex contained channel 1, 2, 3, 5, 17, 18, and 19; the right dorsolateral prefrontal cortex contained channel 4, 6, 21, 23, 40, 41, and 42; the left pars triangularis Broca’s area contained channel 31, 32, 46, and 47; the left pre-motor and supplementary motor cortex contained channel 36 and 48; the left temporal cortex contained channel 13, 14, 15, 16, 33, 34, and 35; the left dorsolateral prefrontal cortex contained channel 10, 12, 29, 30, 43, 44, and 45.

The channel locations have been described in our previous studies (Figure 2). The participants sat comfortably in a quiet room and remained calm during the 600-s detection period associated with the resting-state task prior to initiating the experiments. The patients moved minimally throughout the procedure. The test environment was rigorously controlled to ensure acoustic isolation, and all external auditory disturbances were eliminated during data acquisition. EEG and fNIRS data were acquired sequentially in a single session. The EEG recording was performed first, followed immediately by the fNIRS recording. The transition time between modalities was standardized, with a median interval of 5 min dedicated to repositioning the participant and setting up the second system. Participants remained in the same controlled environment and continued the same experimental paradigm throughout. This sequential design was implemented to avoid physical interference between the two types of sensors.

Hemoglobin concentrations were measured using a NirScan-6000A multi-channel fNIRS brain imaging device (Danyang Huichuang Medical Equipment Co., Ltd., Huichuang, China). The sampling frequency was 11 Hz, and the wavelengths were 730, 808, and 850 nm; 730 nm and 850 nm were the major and isotopic wavelengths, respectively, for correction, as described previously. We used the FPz channel (10/20 International System) as the center of the middle probe. Thirty-one source-detector probes (15 sources and 16 detectors) with a fixed 3-cm inter-probe distance were placed to cover the bilateral prefrontal cortex (PFC) and temporal cortices. In total, 48 NIRS channels were established.

NIRS data were analyzed using the NirSpark package (V1.7.5, Huichuang, China), and the data were preprocessed as follows: (1) Motion artifacts were corrected using moving standard deviation and cubic spline interpolations; (2) Physiological noise was removed using a band pass filter with cut-off frequencies of 0.01–0.20 Hz; 3) Optical densities were converted into altered HbO and HbR concentrations using the modified Beer–Lambert law. We used HbO as the primary indicator in the subsequent analyses instead of HbR owing to its higher signal-to-noise ratio.

### 2.5 Machine learning

We selected the support vector machine (SVM) algorithm for our EEG-based machine learning model. Each feature parameter was individually standardized using a Z-score transformation to eliminate differences in the units of measurement among the feature parameters. In other words, for each feature from each participant, the mean value of that feature for all participants was subtracted and then divided by the standard deviation of that feature across the participants. A linear kernel and a penalty coefficient C of 10 were identified as the optimal hyperparameters for SVM modeling.

We also selected an SVM algorithm for the fNIRS-based machine learning model. Each feature parameter was individually standardized using a Z-score transformation, as described for the EEG model, to eliminate differences in units of measurement. An RBF kernel (Gaussian kernel) and a penalty coefficient C of 10 were identified as the optimal hyperparameters for SVM modeling.

Finally, the SVM algorithm was selected for the combined EEG-fNIRS machine learning model. Each feature parameter was individually standardized using a Z-score transformation, as previously described. A linear kernel and a penalty coefficient C of 10 were identified as the optimal hyperparameters for SVM modeling.

A k-fold cross-validation scheme was employed to evaluate model performance. For each fold, the PCA transformation and feature selection were fitted exclusively on the training data. The resulting transformation and feature subset were subsequently applied to the held-out test data.

We have performed a permutation test with 1,000 iterations to rigorously evaluate the statistical significance of our model’s performance. In each iteration the class labels were randomly shuffled, and the entire model training and evaluation pipeline was repeated.

### 2.6 Statistical analyses

Data are presented as means ± standard deviations. Demographic and clinical variables, as well as HbO changes (representing cortical activation differences), were compared between the AD and LLD groups using Student’s *t*-tests. We have applied the false discovery rate (FDR) correction with a significance threshold of *q* < 0.05 to all channel-wise *t*-tests. Functional connectivity was analyzed using the Pearson’s correlation coefficient between the time series of each channel-to-channel pair. Correlations between HbO variables and blood biomarkers were also analyzed using Pearson’s correlations. Age and sex were incorporated as covariates in our classification. *P* < 0.05 were considered statistically significant. All data were analyzed using IBM SPSS Statistics for Windows version 19.0 (IBM Corp., Armonk, NY, United States).

## 3 Results

### 3.1 Demographic and clinical data

The study included 61, 28, and 37 patients with AD, MCI, and LLD, respectively. Table 1 presents their demographic and clinical characteristics. Age, sex, and education did not differ between the groups (*p* > 0.05). The mean total MMSE score was significantly lower (*p* < 0.05) for patients with AD (14.28 ± 5.60, *p <* 0.001) than for those with MCI (26.86 ± 1.96) and LLD (26.86 ± 1.96). The mean HAMD score was significantly higher (*p* < 0.05) for patients with LLD (10.81 ± 3.79, *p <* 0.001) than for patients with AD (0.23 ± 0.49) and MCI (0.23 ± 0.49). The average age of AD, MCI, and LLD were 75.34 ± 8.22, 71.04 ± 9.08, and 73.81 ± 6.26, respectively.

**TABLE 1:** Demographic and clinical parameters.

Items	AD (*n* = 61)	MCI (*n* = 28)	LLD (*n* = 27)	*F*	*p*-value
Age	75.34 ± 8.22	71.04 ± 9.08	73.81 ± 6.26	2.763	0.067
Gender (f:m)	42:19	21:7	22:5	0.78	0.461
Education	7.13 ± 3.37	7.64 ± 3.28	6.70 ± 2.63	0.599	0.551
MMSE score	14.28 ± 5.60	26.86 ± 1.96	29.33 ± 0.73	138.847	0.000
HAMD score	0.23 ± 0.49	2.71 ± 1.82	10.81 ± 3.79	249.040	0.000

Data are presented as means ± standard deviations. AD, Alzheimer’s disease; MCI, mild cognitive impairment; LLD, late-life depression; MMSE, Mini-Mental State Examination; HAMD, Hamilton Depression Scale.

### 3.2 EEG analyses

#### 3.2.1 EEG power

**FIGURE 3:**
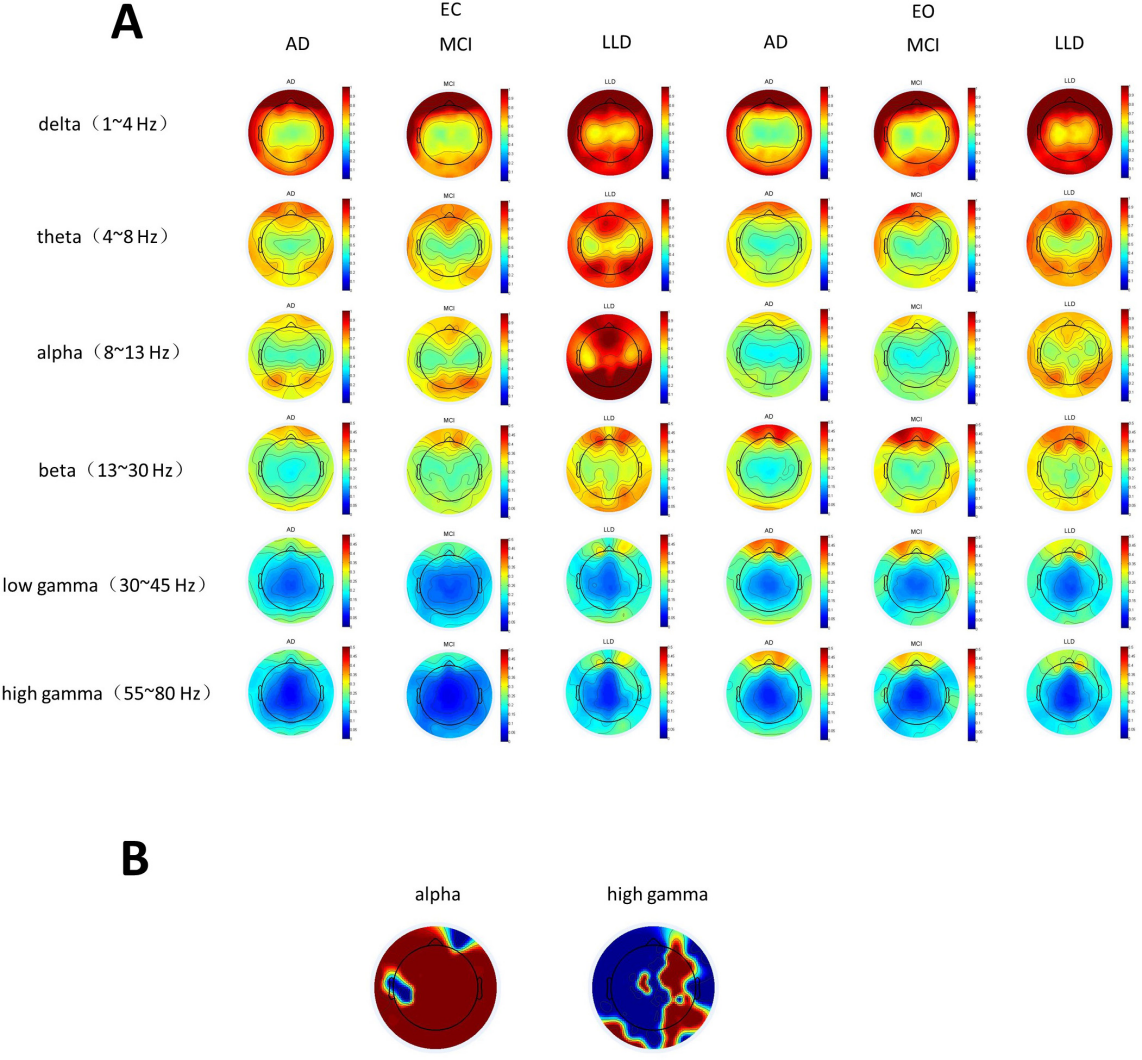
**(A)** Delta, theta, alpha, beta, low gamma, and high gamma band powers from electroencephalography. **(B)** Electrode scalp distributions in the alpha and high gamma frequency bands. EC, eyes closed; EO, eyes open; AD, Alzheimer’s disease; LLD, late-life depression; MCI, mild cognitive impairment.

Alpha band power significantly differed among the three groups in the eyes-closed condition (Figure 3A). Specifically, the alpha band power was significantly higher in the LLD group compared to the AD and MCI groups; however, it did not differ between the AD and MCI groups. The high gamma band power also significantly differed among the three groups. Similarly, the high gamma band power was significantly higher (*p* < 0.05) in the LLD group than in the MCI group, but it did not differ between the AD and MCI groups, nor between the AD and LLD groups. None of the electrode groups for any frequency band differed among the AD, LLD, and MCI groups in the eyes-open condition (Figure 3A). Figure 3B presents the scalp distributions of the electrodes, showing significant differences in the alpha and gamma frequency bands.

#### 3.2.2 EEG wPLI

In the eyes-closed condition, wPLI did not differ among the three groups across all six frequency bands. In the eyes-open condition, the wPLI beta band significantly differed for 242 channel pairs. Specifically, the wPLI was significantly higher (*p* < 0.05) in the MCI group than in the AD group for the 242 channel pairs.

### 3.3 fNIRS analysis of various groups

**FIGURE 4:**
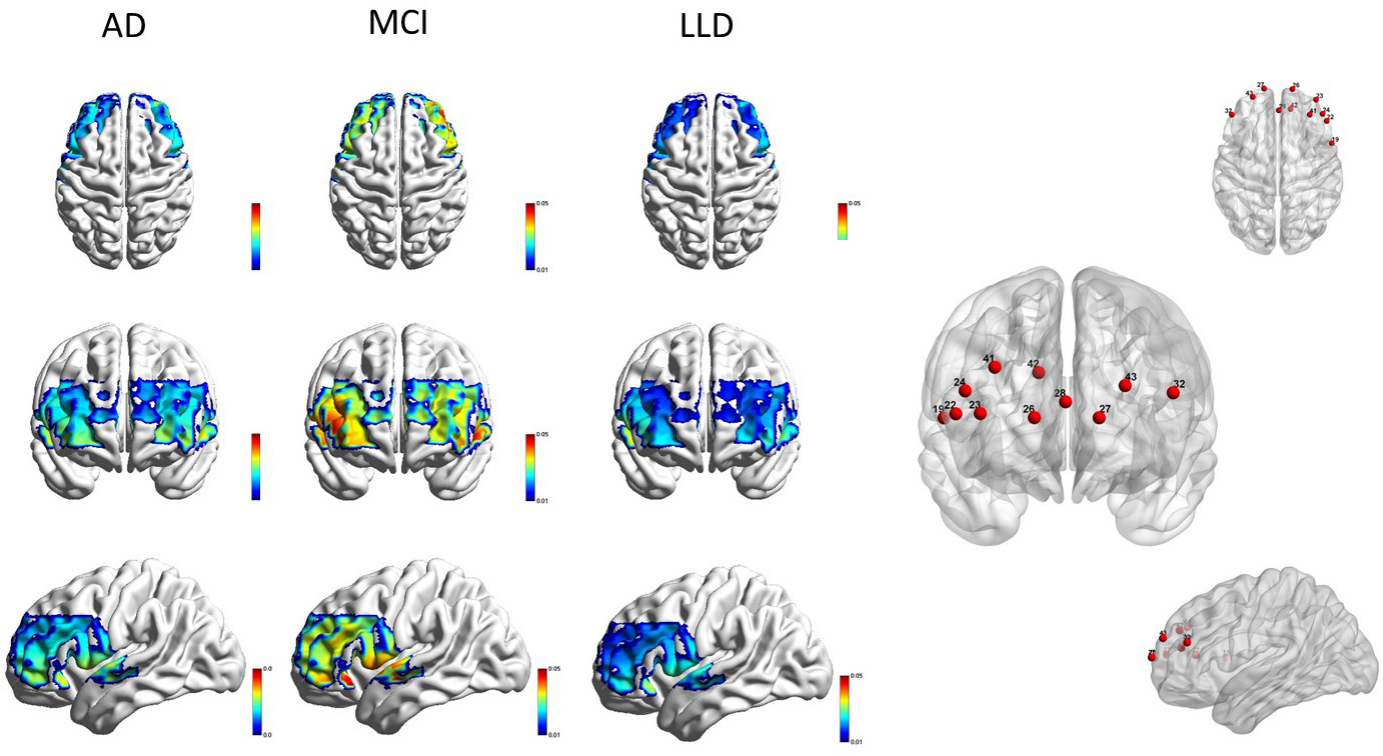
HbO power in patients with AD, LLD, and MCI. The HbO power is significantly lower in the AD group than in the MCI group, but significantly higher in the MCI group than in the LLD group. HbO, oxygenated hemoglobin; AD, Alzheimer’s disease; LLD, late-life depression; MCI, mild cognitive impairment.

Significant differences in HbO power were observed in 11 channels (channels 19, 22, 23, 24, 26, 27, 28, 32, 41, 42, and 43) (Figure 4). Post-hoc comparisons revealed that the values were significantly lower (*p* < 0.05) in the AD group compared to the MCI group and significantly higher (*p* < 0.05) in the MCI group compared to the LLD group.

**FIGURE 5:**
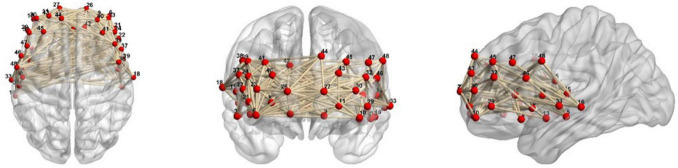
Oxygenated hemoglobin correlation coefficients among the Alzheimer’s disease, late-life depression, and mild cognitive impairment groups are shown in the sagittal, coronal, and horizontal planes.

The correlation coefficients of the 295 channel pairs also significantly differed among the groups. *Post-hoc* analyses revealed significantly higher (*p* < 0.05) correlation coefficients in the MCI group compared to the AD group, but significantly lower (*p* < 0.05) values in the LLD group compared to the MCI group (Figure 5).

### 3.4 EEG and fNIRS prediction models

#### 3.4.1 EEG performance evaluation

A principal component analysis was performed on the power values of the electrodes, revealing significant differences in the alpha and high gamma bands in the eyes-closed condition. Additionally, the wPLI values of the electrode pairs showed significant differences in the beta band in the eyes-open condition. The principal components that cumulatively explained more than 90% of the total variance were retained, resulting in three principal components for eyes-closed alpha power, nine for eyes-closed high gamma power, and 30 for eyes-open beta power. These principal components were combined to extract 42 features for the EEG machine learning model. The sensitivity, specificity, precision, F1 score were 0.4028, 0.5888, 0.4156, and 0.4063, respectively. Ultimately, the SVM model achieved an accuracy of 0.5246 (*p* = 0.0035 < 0.01) for classifying AD, LLD, and MCI.

#### 3.4.2 fNIRS performance evaluation

A principal component analysis was performed on the power values of channels with significant differences in the power analysis, as well as on the correlation coefficients of channel pairs with significant differences in the functional connectivity analysis. The principal components that cumulatively explained more than 90% of the total variance were retained, resulting in five principal components for power and 45 for the correlation coefficient. These principal components were combined to extract 50 features for the fNIRS machine learning model. The sensitivity, specificity, precision, F1 score were 0.3980, 0.6219, 0.3880, and 0.3926, respectively. Ultimately, the SVM model achieved an accuracy of 0.5902 (*p* = 0.0035 < 0.01) for classifying the three diseases.

#### 3.4.3 EEG–fNIRS performance evaluation

The 42 EEG features and 50 fNIRS features were combined, yielding a total of 92 features. The sensitivity, specificity, precision, F1 score were 0.3934, 0.6024, 0.3860, and 0.3980, respectively. Ultimately, this SVM model achieved an accuracy of 0.6066 (*p* = 0.0045 < 0.01) for classifying the three diseases.

## 4 Discussion

Traditional diagnostic tools, such as self-reporting symptoms, primarily rely on subjective assessments. However, biomarkers for diagnosing neuropsychiatric diseases, including those from magnetic resonance imaging and positron emission tomography-computed tomography, have been reported (Davies-Jenkins et al., 2024). fNIRS and EEG have many advantages in diagnosing neuropsychiatric disorders. First, EEG excels in capturing real-time neural activity with millisecond temporal resolution, making it ideal for detecting dynamic brain dysfunction in conditions such as AD, MCI, and schizophrenia. Furthermore, its portability and low cost enable widespread clinical use, particularly for monitoring seizures and sleep disorders (Azami et al., 2023; Jiao et al., 2023). Second, fNIRS complements EEG by measuring hemodynamic changes with good spatial resolution, which is suitable for assessing PFC dysfunction in patients with depression and autism. It is also motion-tolerant, allowing natural movement during tasks, which is advantageous for pediatric or geriatric populations (Owens et al., 2024; Talamonti et al., 2022; Wang et al., 2022). Finally, the integration of EEG and fNIRS offers a multimodal approach that combines the temporal and spatial strengths to enhance the diagnostic accuracy for neuropsychiatric diseases while balancing practicality and patient comfort (Gallego-Molina et al., 2025).

EEG-fNIRS integration enhances the diagnostic accuracy for neuropsychiatric disorders using machine learning (Boby and Veerasingam, 2025; Bomatter et al., 2024). By combining EEG (temporal precision) and fNIRS (hemodynamic spatial resolution), researchers can acquire high-dimensional neural data that reflect brain dysfunction in neuropsychiatric diseases. Furthermore, by leveraging machine learning, these multimodal biomarkers enable the development of highly accurate diagnostic models (Waschkies et al., 2023), and such models have achieved robust performances in binary classification tasks (Deslandes et al., 2004). In this study, we focused on the more challenging three-way classification of AD, LLD, and MCI, finding that the integrated EEG-fNIRS model outperformed the unimodal (i.e., EEG or fNIRS only) approaches, yielding higher diagnostic accuracy. This underscores the synergistic value of multimodal neuroimaging in complex differential diagnoses.

**FIGURE 6:**
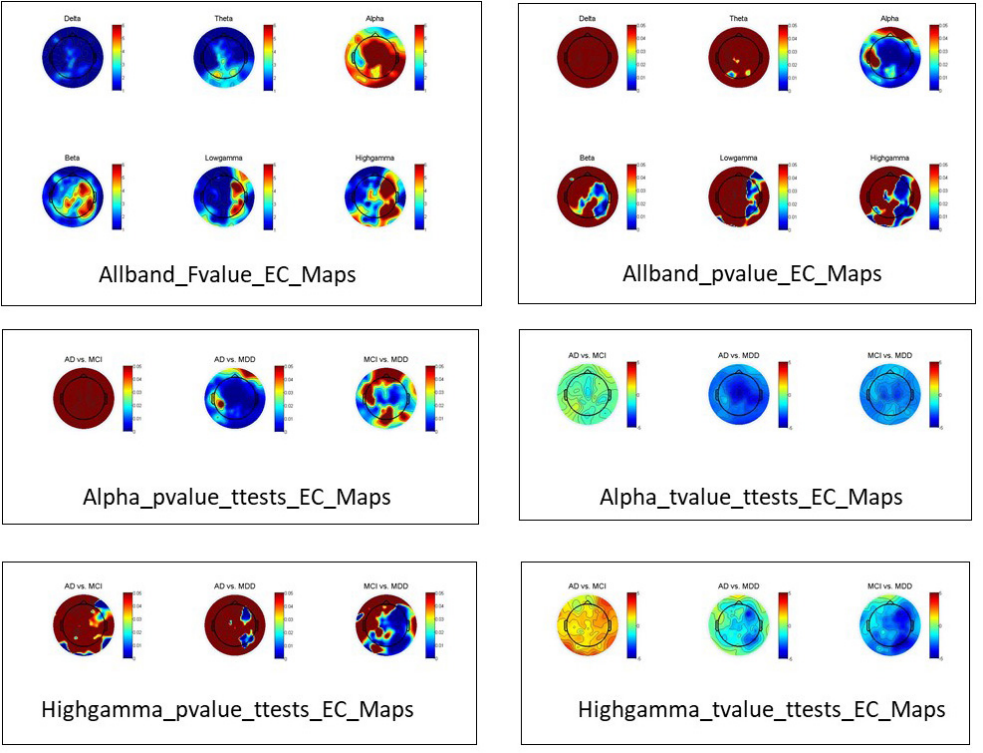
Allband_Fvalue_EC_Maps are topographic maps of the *F*-values from ANOVA performed on power across the three subject groups for each frequency band under the eyes-closed (EC) condition. Allband_pvalue_EC_Maps are topographic maps of the corresponding *p*-values from the same ANOVA analysis for each frequency band. Since the statistical test results indicated significant differences in power among the three groups specifically in the alpha and high-gamma bands under the EC condition, pairwise independent samples *t*-tests were conducted for these two frequency bands. Alpha_pvalue_ttests_EC_Maps and Alpha_tvalue_ttests_EC_Maps are the *p*-value and *t*-value topographic maps, respectively, from the pairwise comparisons (*t*-tests) among the three groups in the alpha band. Highgamma_pvalue_ttests_EC_Maps and Highgamma_tvalue_ttests_EC_Maps are the *p*-value and *t*-value topographic maps, respectively, from the pairwise comparisons (*t*-tests) among the three groups in the high-gamma band.

Our EEG analyses revealed distinct patterns of oscillatory activity and functional connectivity that differentiate LLD) from AD and MCI, particularly under the eyes-closed resting state. The most pronounced finding was the significantly elevated alpha and high-gamma band power in the LLD group compared to both AD and MCI groups in the eyes-closed condition (Figure 6). This result stands in contrast to the typical EEG profile of AD/MCI, which is often characterized by a slowing of the posterior dominant rhythm (alpha reduction) and decreased fast-frequency power as disease progresses (Babiloni et al., 2021; Chetty et al., 2024). LLD may be associated with a state of cortical hyperarousal or inefficient inhibitory processing (Xie et al., 2024). Increased alpha power in depression has been previously interpreted as reflecting excessive internal attention or rumination, a cognitive state prevalent in LLD (Wang et al., 2024). The elevated high-gamma power may indicate heightened neuronal excitability and increased metabolic demand, possibly representing a compensatory cognitive effort to maintain function in the face of depressive symptomatology. The fact that these power differences vanished in the eyes-open condition further underscores their state-dependent nature and suggests that the hyperarousal signature in LLD may be most detectable when external visual input is minimized, allowing internal pathological processes to dominate the EEG signal.

**FIGURE 7:**
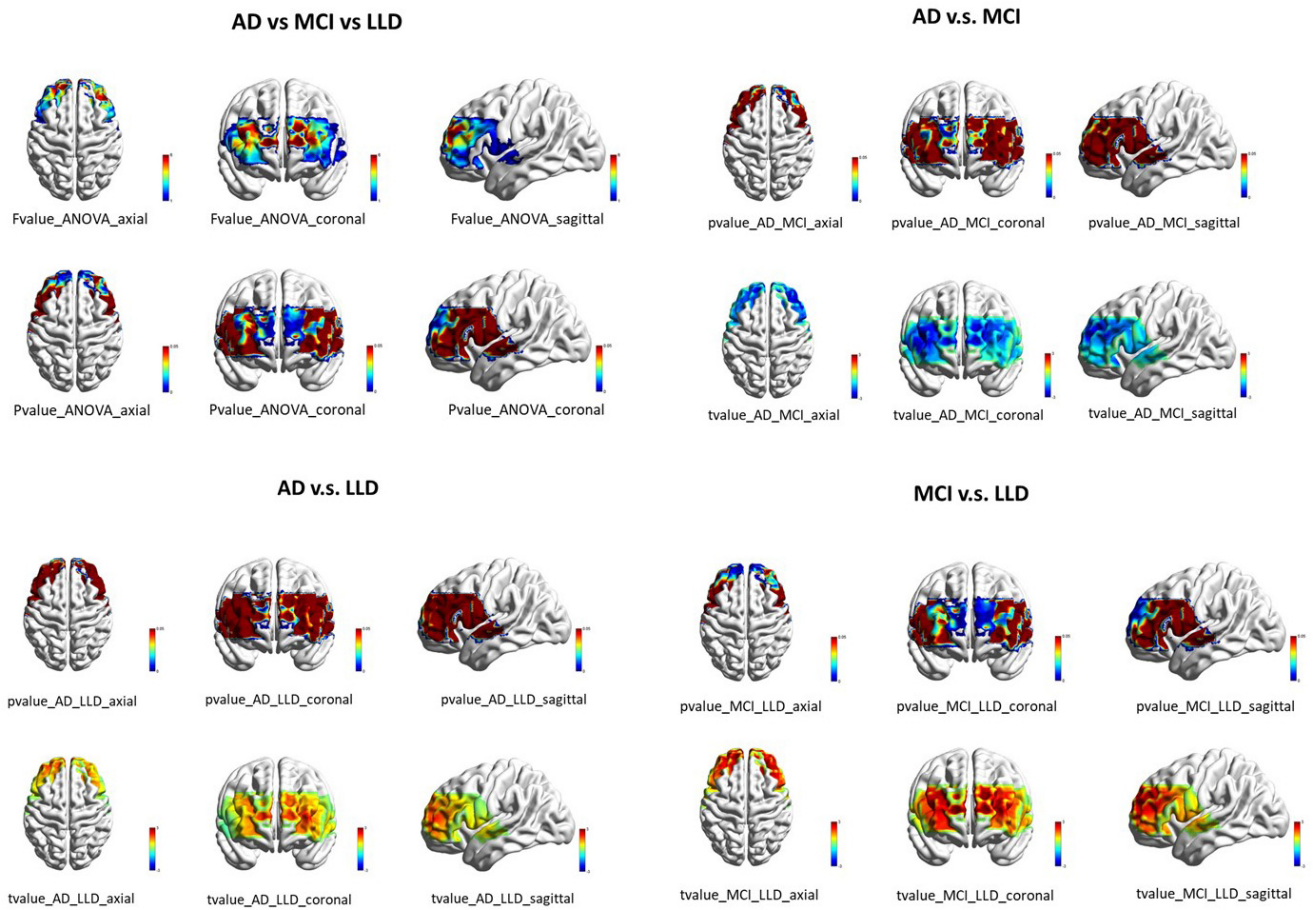
The top-left panel shows *F*-value and *P*-value brain maps from an ANOVA of HbO power across the three groups. The remaining panels display *t*-value and *P*-value brain maps from pairwise group comparisons of HbO power (three views).

Older individuals experiencing LLD are more prone to MCI compared to depressed individuals less than 60 years of age, particularly in memory, attention, and executive function. In the LLD group, the EEG power changes were greater in the right PFC than in the left PFC, which corresponded to HbO changes in the right PFC region on fNIRS. Previous studies have identified microstructural abnormalities in the white matter tracts connecting the PFC with the subcortical and posterior cortical regions in LLD, which are associated with executive dysfunction (Alexopoulos et al., 2009; Bae et al., 2006). However, executive function in AD and MCI are lower than that in LLD (Chu C. et al., 2023). Individuals with LLD and AD frequently experience similar brain alterations, including microvascular diseases, which may play a role in their progression (Soennesyn et al., 2012). Improvements in depressive symptoms can lead to better cognitive function in young patients with major depressive disorder, but executive function deficits often persist in those with LLD (Semkovska et al., 2019). The MCI group demonstrated the highest values of HbO, significantly surpassing both the AD group (lower) and, notably, the LLD group (also lower) (Figure 7). This pattern is highly indicative of a compensatory hyperactivation mechanism in MCI. Prefrontal regions as covered by our fNIRS channels are critical for executive function and working memory. In the prodromal stage of AD (MCI), the brain may recruit additional neural resources and enhance prefrontal hemodynamic activity to maintain cognitive performance despite underlying pathology.

While SVM classifiers have been widely applied in neurodegenerative and psychiatric disorders, their use has been primarily focused on binary classification tasks, such as distinguishing AD from healthy controls or MCI from AD (Chu K. T. et al., 2023; Lee et al., 2022; Wang et al., 2023). The direct three-way classification between AD, MCI, and LLD represents a more clinically pertinent but understudied challenge. Our work advances this area by implementing a tri-class SVM framework based on a novel multi-modal feature set from EEG and fNIRS. The achieved accuracy of 60% underscores the feasibility of this approach. Compared to prior binary studies, our model’s specific pattern of misclassifications provides indirect validation that the selected features capture the critical neurobiological distinction between depressive and neurodegenerative pathophysiology. This multi-modal, electrophysiological-hemodynamic approach appears promising for tackling the complex differential diagnosis in elderly patients with cognitive complaints.

The primary clinical challenge is the significant symptom overlap (e.g., cognitive complaints, apathy) among these conditions, which often leads to diagnostic uncertainty, especially in primary care or memory clinic settings where specialized biomarkers (amyloid-PET, CSF) are not routinely available. Our classifier, based on direct measures of brain function (EEG oscillations) and neurovascular coupling (fNIRS), offers a complementary biological perspective. However, this study has several limitations. First, EEGs were performed before fNIRS, not simultaneously. The temporal gap between EEG and fNIRS is a limitation, as differences in the physiological state, attention, or external conditions of the participants between sessions may have influenced the results, thereby reducing comparability. Second, the sample size and diversity of our study population (e.g., the age, sex, or disease severity of the participants) could be improved to increase the generalizability of the findings to broader populations. Third, performing the EEG first may have introduced practice or fatigue effects that affected the subsequent fNIRS measurements, or vice versa, if counterbalancing was not used. Fourth, differences in the signal-to-noise ratio, motion artifacts, or environmental interference between the EEG and fNIRS sessions could affect data consistency. Fifth, our comparative analysis was conducted using a single classification algorithm SVM. Although SVM is a powerful and widely validated model for structured data, future work should validate these findings across a broader spectrum of classifiers, including interpretable models like regularized logistic regression, robust ensemble methods like Random Forest, and advanced models such as TabPFN. This would help ascertain whether the observed superiority of multimodal fusion is algorithm-agnostic and further optimize the classification framework. Finally, since the two modalities were not synchronized, the potential benefits of integrating EEG and fNIRS data in real time (e.g., improved classification accuracy) were not explored. It is important to note that the classification accuracy achieved here, while promising, is at a proof-of-concept level and falls short of the performance required for direct clinical application.

## 5 Conclusion

This study highlights the distinct EEG spectral patterns of patients with LLD compared to those with AD or MCI, particularly in terms of alpha and high gamma oscillations. These changes could be potential biomarkers for differentiating these three disorders. Combining EEG and fNIRS analyses with machine-learning models may further clarify the neurophysiological mechanisms underlying these disorders.

## Data Availability

The raw data supporting the conclusions of this article will be made available by the authors, without undue reservation.
